# Choroid Plexus Microstructural Alterations Link Glymphatic Dysfunction and White Matter Degeneration in Parkinson's Disease

**DOI:** 10.1002/cns.71039

**Published:** 2026-09-06

**Authors:** Suyi Zhou, Zhiming Zhen, Cheng Lai, Peiyu Huang, Zhe Sun, Taotao Yang, Fengwei Yu, Wei Chen, Zhentao Zuo, Wei Chen

**Affiliations:** ^1^ 7T Magnetic Resonance Imaging Translational Medical Center, Department of Radiology, Southwest Hospital Third Military Medical University (Army Medical University) Chongqing China; ^2^ National Key Laboratory of Cognitive Science and Mental Health, Institute of Biophysics Chinese Academy of Sciences Beijing China; ^3^ University of Chinese Academy of Sciences, Chinese Academy of Sciences Beijing China; ^4^ National Multimode Trans‐Scale Biomedical Imaging Center, Institute of Biophysics Chinese Academy of Sciences Beijing China; ^5^ Interdisciplinary Research Platform for Brain Cognitive Mechanisms and Brain‐Machine Integration, Institute of Biophysics Chinese Academy of Sciences Beijing China; ^6^ Department of Radiology The Second Affiliated Hospital of Zhejiang University School of Medicine Hangzhou China; ^7^ MR Research Collaboration Team, Siemens Healthineers Ltd. Guangzhou China

**Keywords:** choroid plexus, diffusion tensor imaging, glymphatic dysfunction, microstructural changes, Parkinson's disease

## Abstract

**Objectives:**

Glymphatic dysfunction is increasingly recognized as a key contributor to the progression of Parkinson's disease (PD). In PD, both choroid plexus (CP) alterations and white matter abnormalities have been linked to glymphatic dysfunction. However, how CP microstructural abnormalities relate to glymphatic function, corpus callosum (CC) degeneration, and clinical impairment remains unclear. This study aimed to investigate these associations in PD.

**Methods:**

Eighty‐one patients with idiopathic PD and eighty age‐ and sex‐matched healthy controls (HC) underwent MRI scanning. CP microstructural metrics (free water [FW], FW‐corrected fractional anisotropy [FA_FWcorr_] and FW‐corrected mean diffusivity [MD_FWcorr_]) were extracted, along with the diffusion tensor image analysis along the perivascular space (DTI‐ALPS) index and CC subregional volumes. Group comparisons, partial correlation, multiple linear regression, and mediation analyses were performed.

**Results:**

Patients with PD showed significantly higher CP FW and MD_FWcorr_ values and a lower DTI‐ALPS index than HC. Higher CP MD_FWcorr_ was associated with worse motor symptoms and poorer cognitive performance after covariate adjustment. Mediation analysis suggested that the ALPS index mediated the associations between CP microstructural alterations and CC subregional volumes.

**Conclusions:**

CP microstructural abnormalities were associated with DTI‐ALPS alterations, CC degeneration, and clinical impairment in PD. FW‐DTI metrics of the CP may provide useful imaging markers for characterizing CP‐related changes in PD.

## Graphical abstract

This study investigates the role of choroid plexus (CP) microstructural alterations in Parkinson's disease (PD), using free‐water corrected diffusion tensor imaging (FW‐DTI). Elevated CP diffusion metrics were linked to glymphatic dysfunction, corpus callosum degeneration, and worsened motor and cognitive performance in PD patients. These findings highlight CP microstructure as a potential biomarker for PD progression.

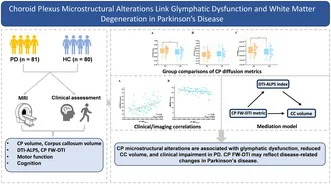

## 1 Introduction

Parkinson's disease (PD) is the second most common neurodegenerative disorder after Alzheimer's disease (AD), characterized by progressive loss of dopaminergic neurons and the abnormal accumulation of α‐synuclein [1]. In addition to classical neurodegenerative features, growing evidence suggests that impaired brain waste‐clearance pathways, particularly dysfunction of the glymphatic system, may underlie or exacerbate pathological progression of PD [2]. The glymphatic system, which enables efficient exchange between cerebrospinal fluid (CSF) and interstitial fluid, plays a pivotal role in clearing neurotoxic solutes from the brain parenchyma, including proteins such as α‐synuclein [3]. Dysfunction of this system may hinder the clearance of α‐synuclein and other metabolic waste products, thereby promoting protein aggregation, triggering neuroinflammation, and accelerating disease progression [4, 5]. Extensive research on cerebral small vessel disease (CSVD) and AD has also highlighted the crucial role of glymphatic dysfunction in white matter damage, which is also closely associated with a range of clinical manifestations in patients [6, 7]. Given the similar pathological mechanisms among neurodegenerative proteinopathies, glymphatic dysfunction has increasingly become a focus of research in PD in recent years. However, the relationship between glymphatic dysfunction, structural brain damage, and motor and cognitive deficits in PD remains poorly understood.

The choroid plexus (CP) is the primary source of CSF and plays a crucial role in maintaining brain immune homeostasis. CP dysfunction is hypothesized to play a critical role in glymphatic impairment. Mechanistically, impaired CP function could diminish the efficiency of CSF production, resulting in reduced CSF flow velocity and subsequent glymphatic transport stasis [8]. Furthermore, increased blood‐cerebrospinal fluid barrier (BCSFB) permeability may allow inflammatory mediators to infiltrate the CSF [9], triggering neuroinflammation that potentially disrupts astrocytic AQP4 polarization [10, 11]. These collective processes impair waste clearance pathways, leading to a systemic decline in glymphatic function.

In PD, CP volume has been shown to negatively correlate with CSF α‐synuclein levels [12], to predict freezing of gait (FOG) through CP area‐to‐lateral ventricle area ratios [13], and to be associated with accelerated cognitive decline [14]. These findings suggest that CP abnormalities may not be isolated lesions, but rather represent a key structural correlate linked to widespread glymphatic dysfunction. On one hand, CP along with imaging markers like perivascular space (PVS) volume and diffusion tensor imaging analysis along the perivascular space (DTI‐ALPS) index is associated with glymphatic degeneration and serves as an indicative neuroimaging biomarker for PD pathogenesis and levodopa response prediction [15, 16, 17]. On the other hand, evaluating brain white matter damage patterns mediated by glymphatic related structures like CP is a key aspect of PD pathophysiology research [18].

The corpus callosum (CC), the largest white matter commissure in the brain, is essential for interhemispheric information integration, motor coordination, and higher cognitive processes [19, 20, 21]. Anatomically, it is a periventricular structure located immediately adjacent to the body and atrium of the lateral ventricles, while the CP projects into these ventricular compartments [22]. Although the CC is not directly continuous with the CP, both structures share the same ventricular microenvironment. In the adjacent deep white matter, perivascular spaces surrounding medullary vessels participate in CSF–ISF exchange [23, 24]. Therefore, CP abnormalities may be associated with changes in the ventricular–perivascular milieu, which may in turn contribute to CC vulnerability.

Volumetric assessments alone may not adequately reflect the subtle microstructural damage of CP in PD. Although DTI metrics are traditionally evaluated in white matter, some studies have also used them as means to assess the microstructural integrity of non‐neural tissues like the CP [25, 26]. Free‐water (FW)‐corrected DTI, which uses a bi‐tensor model to separate the diffusion characteristics of brain tissue from the surrounding free water, provides FW volume fraction and FW‐corrected DTI parameters (e.g., FW‐corrected mean diffusivity [MD_FWcorr_] and FW‐corrected fractional anisotropy [FA_FWcorr_]) [27]. Given that the CP is located within the ventricular system and is susceptible to CSF‐related partial volume effects, FW modeling may help improve the specificity of diffusion estimates within this structure and provide additional insights into CP‐related microstructural alterations in PD. Therefore, we hypothesized that CP microstructural abnormalities are associated with glymphatic dysfunction in PD and are related to CC degeneration and clinical impairment. The primary objectives of this study are: (1) to investigate CP microstructural changes in patients with PD using FW‐DTI; (2) to evaluate the associations between these CP alterations and the DTI‐ALPS index, CC volume, and clinical motor/cognitive impairment, and (3) to investigate whether the DTI‐ALPS index mediates the association between CP abnormalities and CC atrophy using mediation analysis.

## 2 Materials and Methods

### 2.1 Participants and Clinical Assessments

A total of 90 patients with idiopathic PD and 82 age‐ and sex‐matched healthy controls (HC) were recruited from the First Affiliated Hospital of the Army Medical University between May 2023 and April 2024. The diagnosis of PD was confirmed by two experienced neurologists based on the Movement Disorder Society (MDS) clinical diagnostic criteria for PD [28]. The exclusion criteria were as follows: (1) Patients with parkinsonism secondary to cerebrovascular disease, encephalitis, poisoning, or other neurodegenerative disorders (*n* = 3); (2) datasets lacking diffusion‐weighted imaging or affected by severe MRI artifacts (*n* = 6). HC had no neurologic or systemic disorders that could potentially affect the CNS and showed a completely normal neurologic examination. A total of 161 participants were included in the final analysis. The detailed patient selection process is illustrated in Figure S1.

Neurological examinations were evaluated using Mini‐Mental State Examination (MMSE) [29], Parkinson's Disease Rating Scale (UPDRS part III) [30], and Hoehn and Yahr (HY) stages [31]. All clinical evaluations were performed within 1 week of MRI acquisition by a trained neurologist blinded to imaging results. Patients with PD receiving dopaminergic therapy were examined in the clinically defined “OFF” state, after withholding all dopaminergic medications for at least 12 h.

### 2.2 MRI Acquisition

All imaging data were acquired using a 3T MRI scanner (Trio Tim, Siemens Healthineers, Erlangen, Germany). High‐resolution three‐dimensional sagittal T1‐weighted images were collected using magnetization‐prepared rapid acquisition gradient echo (MPRAGE) sequence with the following parameters: repetition time (TR) = 1900 ms, echo time (TE) = 2.52 ms, inversion time (TI) = 900 ms, matrix size = 256 × 256, flip angle = 9°, field of view (FOV) = 256 × 256 mm^2^, slice thickness = 1 mm without slice gap, voxel size = 1.0 × 1.0 × 1.0 mm, slice number = 176. DTI was acquired using a single‐shot spin‐echo echo‐planar imaging (EPI) sequence: TR = 10,000 ms, TE = 92 ms, FOV = 256 × 256 mm^2^, matrix = 128 × 128, slice thickness = 2 mm without slice gap, voxel size = 2.0 × 2.0 × 2.0 mm, 64 non‐collinear directions at *b* = 1000/2000 s/mm^2^, plus one b0 image.

### 2.3 Volumetric Quantification

T1‐weighted images were processed using FreeSurfer (version 7.3.2; https://surfer.nmr.mgh.harvard.edu/) [32, 33]. The standard automated pipeline was applied to generate whole‐brain segmentation, from which CP volume and CC subregional volumes were extracted. CP segmentations were visually inspected and manually corrected when necessary by two experienced radiologists according to anatomical boundaries; discrepancies were resolved by a third senior radiologist. CC subregional volumes were obtained according to the standard FreeSurfer parcellation scheme, and all segmentations underwent visual quality assessment.

### 2.4 DTI Preprocessing and Quantification

DTI preprocessing was performed using FMRIB Software Library (FSL v6.0.5) and MRtrix3. The pipeline included: correction for eddy currents and head motion using eddy_correct (FSL); skull stripping using BET. Diffusion tensor fitting was performed using FSL DTIFIT based on the *b* = 1000 s/mm^2^ shell only. The higher *b*‐value (*b* = 2000 s/mm^2^) data were excluded from tensor estimation to ensure compatibility with the assumptions of the diffusion tensor model. FA and MD maps were derived from the fitted tensors.

#### 2.4.1 Choroid Plexus Free‐Water Diffusion Analysis

Free‐water correction was performed using the bi‐tensor model proposed by Pasternak et al. [27], which separates the diffusion signal into two compartments: an anisotropic tissue compartment and an isotropic free‐water compartment. Model fitting was implemented following the framework described by Metzler‐Baddeley et al. [34], including regularization to stabilize parameter estimation. Voxel‐wise free‐water fraction and FW‐corrected diffusion tensor metrics, including FA_FWcorr_ and MD_FWcorr_, were derived from the tissue compartment.

#### 2.4.2 DTI‐ALPS Analysis

The DTI‐ALPS index was calculated according to the method described by Taoka et al. [35]. Briefly, 5‐mm cross‐shaped regions of interest (ROIs) were defined using an atlas‐guided approach to localize the projection and association fiber regions adjacent to the lateral ventricles, consistent with previous implementations of the DTI‐ALPS method [36], as shown in Figure S2. The ROI positions were then manually checked for each individual on color‐coded FA maps to ensure accurate placement and to avoid contamination from cerebrospinal fluid or gray matter. The ALPS index was calculated as follows:

$$\text{ALPS index}=\frac{\text{mean}\left(D_{\text{xxproj}},D_{\text{xxassoc}}\right)}{\text{mean}\left(D_{\text{yyproj}},D_{\text{zzassoc}}\right)}$$

The final ALPS index for each participant was calculated as the average of the values obtained from both hemispheres. Additional details of the DTI‐ALPS analysis are provided in Supplementary Method 1.

### 2.5 Statistical Analysis

All statistical analyses were conducted using SPSS Statistics (IBM Corp., Armonk, NY, USA) and OriginPro (version 2022; OriginLab Corp., Northampton, MA, USA). ChiPlot (https://www.chiplot.online/) was used for correlation heat map visualization. Continuous variables were tested for normality using the Shapiro–Wilk test and are presented as mean ± SD or median (IQR), as appropriate; categorical variables are presented as counts (%).

Demographic and clinical characteristics were compared between patients with PD and HC using independent‐samples *t*‐tests, Mann–Whitney *U* tests, or chi‐square tests, as appropriate. CP and CC subregional volumes were corrected for total intracranial volume (TIV) using the residual approach [37]. Group comparisons in imaging measures were assessed using analysis of covariance (ANCOVA), with age, sex, education level, and Hamilton Depression Rating Scale (HAMD) score included as covariates.

Partial correlation analyses were conducted to examine the relationships between CP diffusion metrics, the DTI‐ALPS index, and clinical variables, adjusting for age, sex, education level, HAMD score, levodopa equivalent daily dose (LEDD), and disease duration; analyses involving the DTI‐ALPS index were additionally adjusted for global white matter FA and MD to minimize the influence of generalized white matter microstructural degeneration on the ALPS measure. Mediation analyses were performed using the PROCESS macro for SPSS (Model 4) with 5000 bootstrap samples to test whether the DTI‐ALPS index mediated the association between CP microstructural metrics and CC subregional volumes, with age, sex, and global WM‐FA/MD as covariates. Indirect effects were considered significant when the 95% CI did not include zero.

Multiple linear regression was used to identify independent predictors of motor and cognitive performance. To reduce multicollinearity and overfitting, only CC subregions significant in univariate analyses were entered. Model assumptions were checked, including linearity, residual normality, and multicollinearity (variance inflation factor < 10), and model fit was assessed using adjusted *R* ^2^.

A two‐tailed *p* value < 0.05 was considered statistically significant unless otherwise specified. For group comparisons and correlation analyses, *p* values were adjusted for multiple testing using the Benjamini–Hochberg FDR procedure within each family of related tests, with FDR‐adjusted *p* values (*p*_*FDR*_) < 0.05 considered significant.

## 3 Results

### 3.1 Characteristics and Group Comparison

The PD and HC groups were comparable in terms of age (62.27 ± 7.81 vs. 61.99 ± 6.21 years, *p* = 0.80) and gender distribution (male: 46.91% vs. 38.75%, *p* = 0.30). Patients with PD exhibited significantly higher HAMD scores (13.69 ± 9.04 vs. 3.99 ± 3.71, *p* < 0.001), lower MMSE scores (26.58 ± 2.72 vs. 28.32 ± 1.91, *p* < 0.001), and fewer years of education (9.82 ± 3.46 vs. 12.79 ± 2.80, *p* < 0.001) compared to the HC group. Detailed demographic and clinical characteristics are provided in Table S1.

Compared with HC, patients with PD showed higher CP volume (*F*(1, 156) = 4.12, *p* = 0.044), although this difference did not survive FDR correction (*p*_*FDR*_ = 0.088). For CC subregional volumes, nominal group differences were observed in the central and mid‐anterior segments, with lower volumes in the PD group than in HC; however, these differences did not remain significant after FDR correction (central: *F*(1, 156) = 5.40, *p* = 0.021, *p*_*FDR*_ = 0.063; mid‐anterior: *F*(1, 156) = 5.72, *p* = 0.019, *p*_*FDR*_ = 0.063). No significant group differences were observed in the posterior, mid‐posterior, or anterior CC subregions (*p* > 0.05). For CP diffusion metrics, the PD group showed significantly increased FW (*F* (1, 156) = 4.79, *p* = 0.030, *p*_*FDR*_ = 0.040) and MD_FWcorr_ (*F* (1, 156) = 5.63, *p* = 0.019, *p*_*FDR*_ = 0.038), whereas FA_FWcorr_ did not differ significantly between groups (*F* (1, 156) = 1.20, *p* = 0.274), as illustrated in Figure S3. The DTI‐ALPS index was significantly lower in the PD group (*F* (1, 154) = 5.79, *p* = 0.017, *p*_*FDR*_ = 0.038). All group comparisons were performed using ANCOVA adjusted for age, sex, education level, and HAMD score; analyses involving the DTI‐ALPS index were additionally adjusted for global white matter FA and MD. These findings are summarized in Table 1.

**TABLE 1:** Group comparisons of imaging measures between patients with Parkinson's disease and healthy controls using analysis of covariance (ANCOVA).

	PD (*n* = 81)	HC (*n* = 80)	*F*	*p*_*UNCORR*_	*p*_*FDR*_	Partial *η* ^2^
Volumes (mm^3^)						
CPV	1568.60 ± 62.57	1363.42 ± 63.79	4.12	0.044	0.088	0.026
CC_Posterior_corr	956.19 ± 18.23	977.06 ± 18.58	0.50	0.480	0.576	0.003
CC_Mid_Posterior_corr	462.14 ± 11.46	475.92 ± 11.68	0.55	0.458	0.576	0.004
CC_Central_corr	475.93 ± 13.67	527.30 ± 13.94	5.40	0.021	0.063	0.034
CC_Mid_Anterior_corr	483.52 ± 16.62	547.80 ± 16.94	5.72	0.019	0.063	0.036
CC_Anterior_corr	817.89 ± 19.54	830.34 ± 19.92	0.15	0.694	0.694	0.001
Diffusion parameters						
CP FW	0.88 ± 0.01	0.86 ± 0.01	4.79	0.030	0.040*	0.031
CP FA_FWcorr_	0.17 ± 0.003	0.18 ± 0.003	1.20	0.274	0.274	0.008
CP MD_FWcorr_ (×10^−3^ mm^2^/s)	2.26 ± 0.02	2.18 ± 0.02	5.63	0.019	0.038*	0.036
DTI‐ALPS index	1.43 ± 0.01	1.47 ± 0.01	5.79	0.017	0.038*	0.036

*Note:* Data are presented as estimated marginal means ± standard error. Group comparisons were performed using general linear models with age, sex, education level, and HAMD score included as covariates. For the DTI‐ALPS index, global WM‐FA and WM‐MD were additionally included as covariates to minimize the influence of generalized white matter microstructural degeneration. FDR correction was applied for multiple comparisons. Partial *η* ^2^ is reported as a measure of effect size. *Statistically significant after FDR correction (*p*_*FDR* _ < 0.05).

Abbreviations: CC, corpus callosum; CPV, choroid plexus volume; DTI‐ALPS, diffusion tensor image analysis along the perivascular space; FA_FWcorr_, free water–corrected fractional anisotropy; FW, free water; HC, healthy controls; MD_FWcorr_, free water–corrected mean diffusivity; PD, Parkinson's disease.

### 3.2 Correlation Between Choroid Plexus Microstructure and Brain Volumetric Measures

**FIGURE 1:**
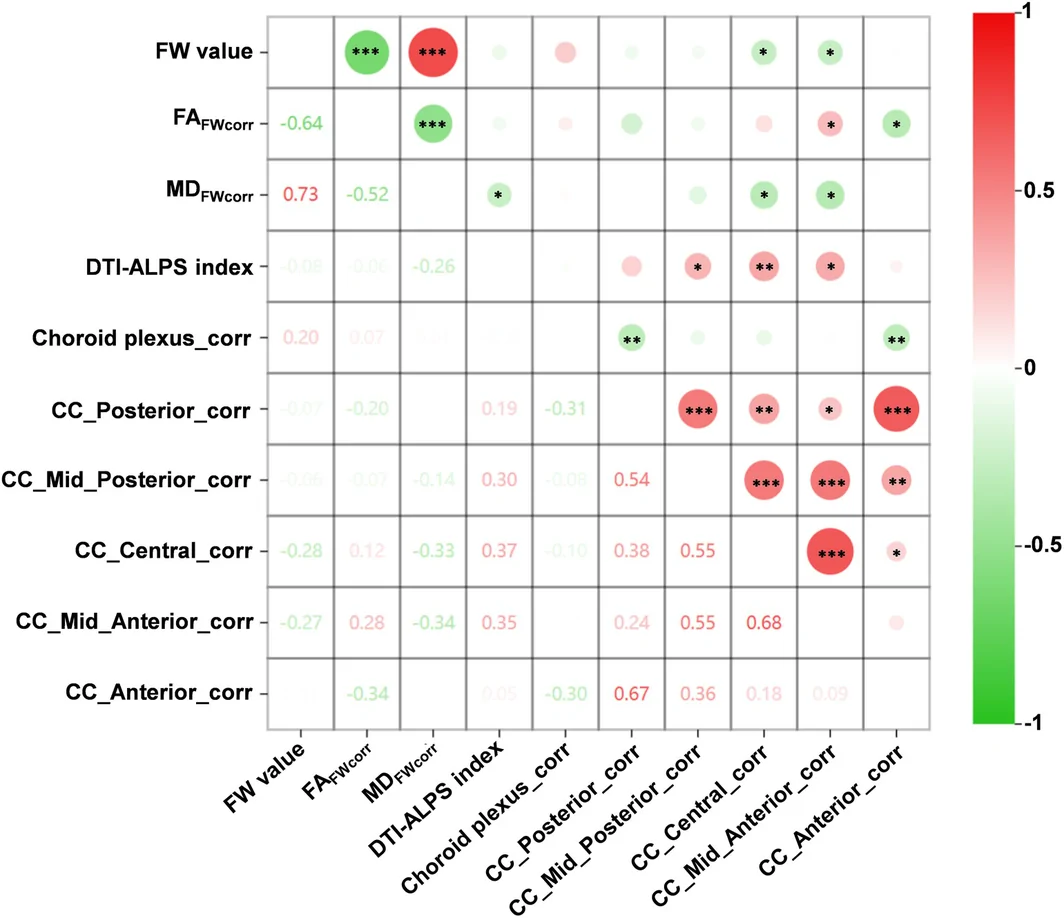
Correlation heatmap of CP diffusion metrics with relevant brain regions. The heatmap illustrates pairwise correlations among choroid plexus diffusion metrics (FW, FA_FWcorr_, and MD_FWcorr_), the DTI‐ALPS index, residual‐corrected choroid plexus volume, and residual‐corrected corpus callosum subregional volumes. Circle color and size represent the direction and magnitude of the correlation coefficients, respectively. Asterisks indicate correlations that remained significant after multiple‐comparison correction. CC, corpus callosum; CP, choroid plexus; DTI‐ALPS, diffusion tensor image analysis along the perivascular space; FA_FWcorr_, free water‐corrected fractional anisotropy; FW, free water; MD_FWcorr_, free water‐corrected mean diffusivity. Variables with the suffix “_corr” denote values adjusted using the residual method.

Partial correlation analysis demonstrated that CP MD_FWcorr_ was significantly negatively correlated with the DTI‐ALPS index and with the central and mid‐anterior subregions of the corpus callosum, and these associations remained significant after multiple‐comparison correction. The full partial correlation matrix is presented in Figure 1.

### 3.3 Associations Between Choroid Plexus Diffusion Metrics and Clinical Measures

**FIGURE 2:**
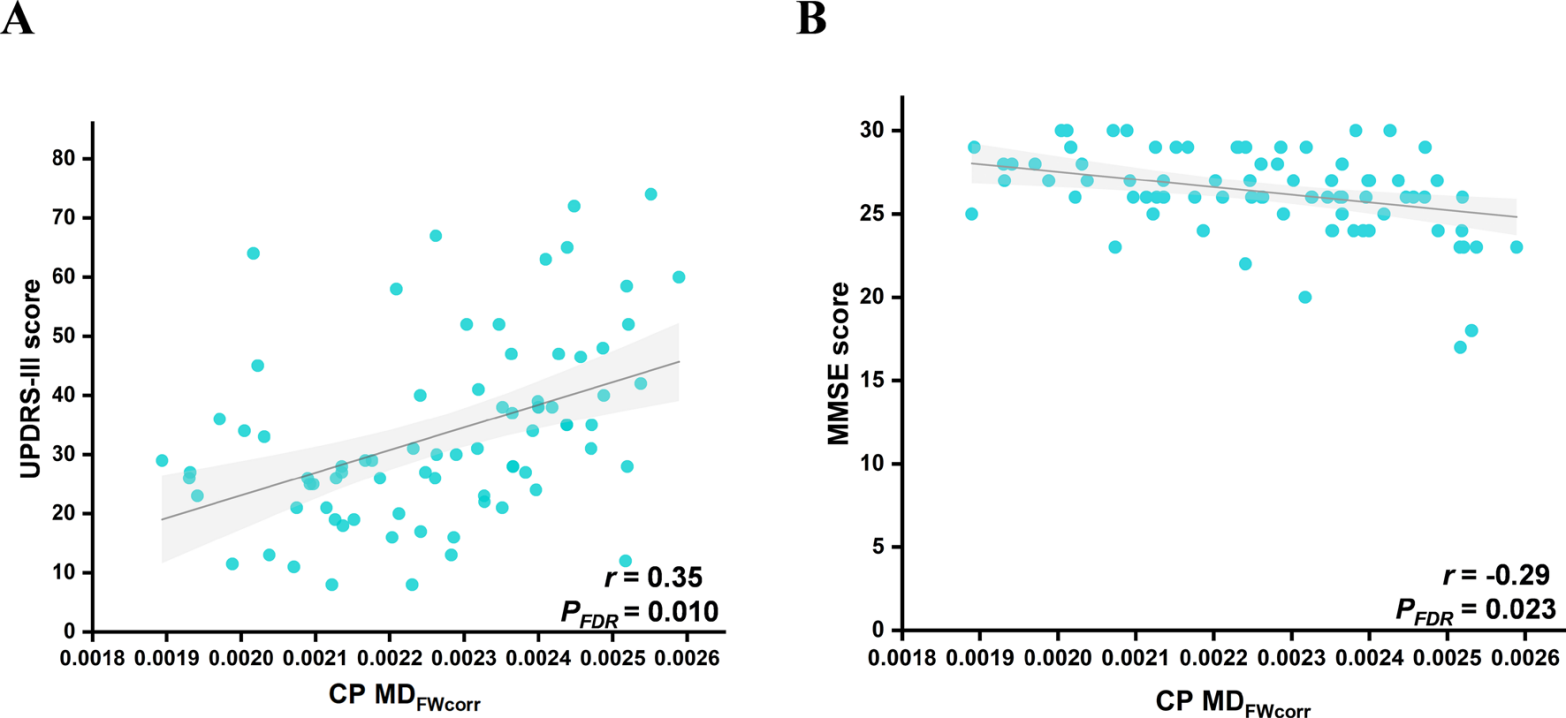
Correlations between choroid plexus diffusion metrics and clinical measures. (A) Positive correlation between CP MD_FWcorr_ and motor symptom severity, assessed by the UPDRS III. (B) Negative correlation between CP MD_FWcorr_ and global cognitive function, measured by the MMSE. CP, choroid plexus; MD_FWcorr_, free water corrected mean diffusivity; MMSE, Mini‐Mental State Examination; UPDRS III, Unified Parkinson's Disease Rating Scale part III.

As demonstrated in Figure 2 and Tables 2 and 3, correlation and regression analyses revealed that CP microstructural alterations are significantly associated with both motor and cognitive dysfunction in PD. CP MD_FWcorr_ showed significantly positive correlations with motor severity (UPDRS III: *r* = 0.35, *p*_*FDR*_ = 0.010) and negative correlations with cognitive function (MMSE: *r* = −0.29, *p*_*FDR*_ = 0.023). Multiple linear regression further confirmed CP MD_FWcorr_ as an independent predictor of both UPDRS III (*β* = 0.387, *p* = 0.024) and MMSE scores (*β* = −0.414, *p* = 0.009), even after adjustment for demographic, clinical, and imaging covariates.

**TABLE 2:** Multiple linear regression analysis of factors associated with MMSE scores in patients with Parkinson's disease.

	*B*	SE	*β*	*t*	*p*
Age	−0.011	0.045	−0.033	−0.248	0.805
Sex	−1.481	0.732	−0.285	−2.023	0.047
Education	0.206	0.089	0.273	2.306	0.024*
Disease duration	−0.165	0.084	−0.264	−1.981	0.052
LEDD	0.001	0.001	0.098	0.758	0.451
HAMD	−0.021	0.037	−0.070	−0.577	0.566
DTI‐ALPS index	−4.211	2.563	−0.214	−1.643	0.105
CPV_corr	−0.084 × 10^−3^	0.001	−0.016	−0.112	0.911
CC_Posterior_corr	0.004	0.002	0.245	1.733	0.088
CP MD_FWcorr_	−6049.271	2226.033	−0.414	−2.718	0.009*

*Note:* Variables with the suffix “_corr” denote values adjusted using the residual method. *Statistically significant after FDR correction (*p_FDR_* < 0.05).

Abbreviations: CC_Posterior_corr, residual‐corrected posterior corpus callosum volume; CP MD_FWcorr_, choroid plexus free water–corrected mean diffusivity; CPV_corr, residual‐corrected choroid plexus volume; DTI‐ALPS, diffusion tensor image analysis along the perivascular space; HAMD, Hamilton Depression Rating Scale; LEDD, levodopa equivalent daily dose; MMSE, Mini‐Mental State Examination.

**TABLE 3:** Multiple linear regression analysis of factors associated with UPDRS III scores in patients with Parkinson's disease.

	*B*	SE	*β*	*t*	*p*
Age	−0.121	0.332	−0.055	−0.365	0.717
Sex	−0.080	5.347	−0.002	−0.015	0.988
Education	0.509	0.612	0.099	0.832	0.410
Disease duration	0.612	0.582	0.150	1.051	0.298
LEDD	0.014	0.010	0.203	1.459	0.151
HAMD	0.499	0.268	0.250	1.862	0.069
DTI‐ALPS	7.819	20.185	0.061	0.387	0.700
CPV_corr	−0.004	0.004	−0.110	−0.874	0.387
CC_Mid_Anterior_corr	−0.002	0.037	−0.011	−0.054	0.957
CC_Central_corr	−0.029	0.029	−0.203	−0.995	0.325
CP MD_FWcorr_	36565.434	15719.496	0.387	2.326	0.024*

*Note:* Variables with the suffix “_corr” denote values adjusted using the residual method. *Statistically significant after FDR correction (*p_FDR_ * < 0.05).

Abbreviations: CC_Central_corr, residual‐corrected central corpus callosum volume; CC_Mid_Anterior_corr, residual‐corrected mid‐anterior corpus callosum volume; CP MD_FWcorr_, choroid plexus free water–corrected mean diffusivity; CPV_corr, residual‐corrected choroid plexus volume; DTI‐ALPS, diffusion tensor image analysis along the perivascular space; HAMD, Hamilton Depression Rating Scale; LEDD, levodopa equivalent daily dose; UPDRS III, Unified Parkinson's Disease Rating Scale Part III.

### 3.4 Mediation Effect of DTI‐ALPS on the Association Between CP Diffusion and Corpus Callosum Volume

**FIGURE 3:**
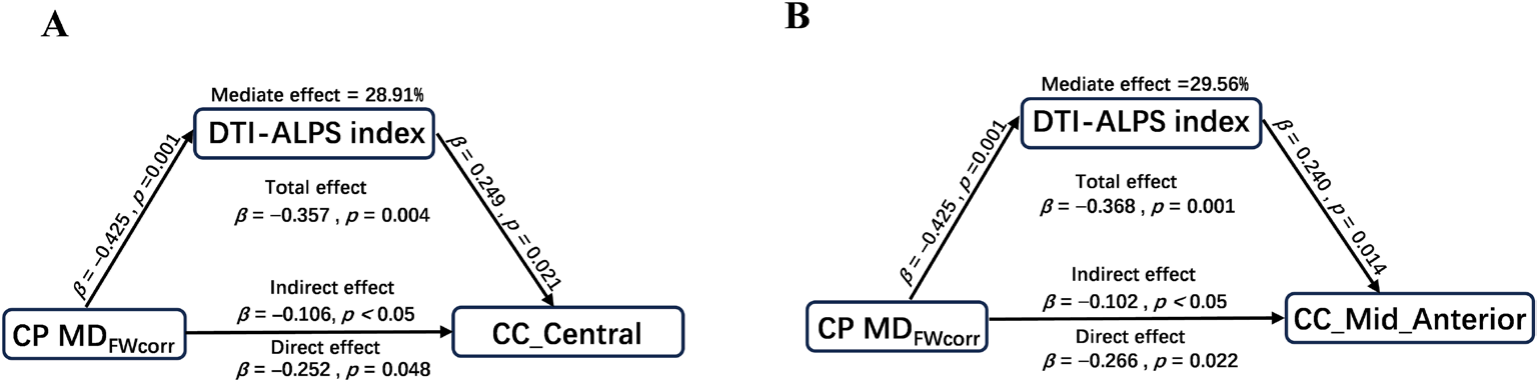
Mediation analysis of the DTI‐ALPS index in the relationship between CP diffusion metrics and subregional CC volumes. The DTI‐ALPS index partially mediated the associations between CP MD_FWcorr_ and residual‐corrected volumes of the central (A) and mid‐anterior (B) corpus callosum subregions. CC, corpus callosum; CP, choroid plexus; DTI‐ALPS, diffusion tensor image analysis along the perivascular space; MD_FWcorr_, free water‐corrected mean diffusivity. Residual‐corrected CC volumes were derived using the residual method.

As illustrated in Figure 3, the DTI‐ALPS index partially mediated the association between CP MD_FWcorr_ and CC subregional volumes after adjustment for age, sex, and whole‐brain white matter FA and MD. Significant indirect effects were observed for the central CC (*β* = −0.106, *p* < 0.05; mediation proportion = 28.91%) and mid‐anterior CC (*β* = −0.102, *p* < 0.05; mediation proportion = 29.56%), indicating partial mediation in these subregions.

## 4 Discussion

To our knowledge, this study is among the first to investigate CP microstructural markers in PD. We further examined their relationships with the DTI‐ALPS index and clinical features. The main findings are summarized as follows. (1) Patients with PD showed significantly increased CP FW and CP MD_FWcorr_ values compared to HC group; (2) Higher CP MD_FWcorr_ values were significantly correlated with more severe motor symptoms (UPDRS III scores) and poorer cognitive performance (MMSE scores). In multivariate regression models, CP MD_FWcorr_ emerged as an independent predictor of both motor and cognitive impairment. (3) The DTI‐ALPS index was significantly reduced in the PD group and mediated the associations between CP MD_FWcorr_ and subregional volumes of the CC.

Before addressing microstructural changes, it is important to acknowledge that the literature on CP macrostructural volume in PD remains inconsistent, with prior studies reporting enlargement, atrophy, or no significant change [9, 38]. In our cohort, CP volume showed only a nominal group difference, which did not survive more stringent correction procedures, including residual‐based adjustment for head size and correction for multiple comparisons. This suggests that macrostructural CP volume differences in PD are modest and method‐dependent, and therefore should be interpreted with caution rather than regarded as a robust structural hallmark of the disease.

In contrast, CP microstructural alterations assessed by FW imaging were more consistent in our dataset, with both CP FW and CP MD_FWcorr_ remaining significantly altered in PD after covariate adjustment and FDR correction. These findings suggest that FW‐based metrics may offer additional insight into CP changes that are not fully captured by macroscopic volumetric measures alone. Accordingly, our results support a complementary role for microstructural assessment in the evaluation of CP involvement in PD.

Our findings revealed that patients with PD exhibited significantly elevated MD_FWcorr_ and FW values in the CP compared to the HC group, suggesting microstructural disruption of this structure. Generally, MD_FWcorr_ reflects the overall diffusivity of water molecules within tissue [39]; higher values generally indicate widespread cellular alterations, including reduced cellularity, edema, or necrosis [40]. The elevated FW fraction suggests extracellular FW accumulation, a common feature in neurodegenerative diseases [41]. In the present study, the observed increase in MD_FWcorr_ may reflect reduced microstructural constraint within the CP. As discussed by Alisch et al. [42], higher diffusivity in the CP has been interpreted as potentially reflecting loss of structural integrity and increased leakiness of epithelial cell junctions. Although the precise mechanisms driving CP alterations in PD remain incompletely understood, accumulating neuropathological evidence implicates proteinopathy and neuroinflammation as major contributors. α‐synuclein aggregation, the pathological hallmark of PD, not only exerts neurotoxicity but also triggers inflammatory and immune responses within the brain [43]. Mollenhauer et al. [44] reported α‐synuclein reactivity in the CP epithelium of post‐mortem PD brains, providing direct evidence for CP involvement in PD‐related pathology. Moreover, Barbariga et al. [45] showed that oxidized and deamidated ceruloplasmin (Cp‐ox/de), a pathological CSF component in PD, impairs proliferation and induces apoptosis of CP epithelial cells. Taken together, these findings support the interpretation that increased CP diffusion metrics in PD reflect underlying microstructural damage driven by proteinopathy and neuroinflammatory processes.

We found that CP MD_FWcorr_ was positively correlated with motor symptom severity (UPDRS III scores) and negatively correlated with cognitive function (MMSE scores) in patients with PD. In multivariable models, CP MD_FWcorr_ remained independently associated with both UPDRS III and MMSE scores after adjustment for demographic, clinical, and volumetric measures of the CP and CC. These findings suggest that CP diffusion‐derived metrics may provide clinically relevant information beyond conventional volumetric measures. Existing evidence, largely based on CP volumetric measures, suggests that CP abnormalities are related to both motor and cognitive impairment in PD. Increased CP volume has been associated with more severe motor deficits, possibly through regional glymphatic dysfunction involving the basal ganglia [16], and with reduced dopamine transporter availability in the posterior putamen, suggesting that CP alterations may influence motor symptoms either directly or through dopaminergic pathways [46]. In addition, CP enlargement has been linked to cognitive decline in early‐stage PD [14], and baseline CP volume has been associated with frontal/executive dysfunction as well as a higher risk of dementia conversion [47]. Our results add to this literature by showing that CP microstructural abnormalities are also associated with clinical manifestations in PD. This may indicate that diffusion‐based CP measures capture pathological changes that are not fully reflected by volume alone.

Our findings extend existing evidence by demonstrating that microstructural disruption of the CP is significantly associated with a lower DTI‐ALPS index, suggesting altered perivascular fluid diffusivity and possible glymphatic‐related disturbance in PD. The specificity of the DTI‐ALPS index as a glymphatic marker remains debated. Some studies have reported supportive evidence for the biological relevance of the ALPS index. For example, Zhang et al. [48] reported a strong correlation (*r* > 0.77) between the ALPS index and intrathecal contrast clearance, while other studies have linked it to CSF biomarkers (Aβ and tau) in AD [49]. Conversely, Storås et al. [50] failed to replicate these findings with contrast‐enhanced MRI, suggesting the index may be influenced by the geometric microstructure of adjacent fiber tracts. Despite these interpretative limitations, the DTI‐ALPS index has shown clinically relevant associations in PD, including correlations with motor severity, cognitive decline, and disease progression [51, 52, 53]. While it does not directly measure “fluid flow,” it may reflect aspects of perivascular and neurovascular microenvironmental change that are relevant to glymphatic‐related processes. We therefore interpret the DTI‐ALPS index as an imaging proxy that may reflect altered fluid diffusivity and homeostatic disturbances within the perivascular space. The significant association observed here between CP alterations and the ALPS index supports the relevance of this metric to perivascular fluid‐related changes in PD, while still warranting cautious interpretation.

Within this interpretative framework, our mediation analysis revealed that the ALPS index significantly mediated the relationship between CP MD_FWcorr_ and the volume of CC subregions. The CC is critical for interhemispheric integration of motor, sensory, and cognitive information [54], and CC alterations represent one of the most consistent white matter abnormalities observed in PD, often emerging in early stages and progressing with disease severity [19]. Additionally, the rich network of deep medullary veins (DMVs) and perivascular channels surrounding the corpus callosum (CC) may be particularly vulnerable to damage and may be associated with white matter degeneration [55]. These findings suggest that CP microstructural abnormalities may be linked with interhemispheric white matter degeneration in relation to glymphatic dysfunction, thus providing a mechanistic link between peripheral CSF‐related pathology and central neural architecture.

There are several limitations to this study. First, the automatic segmentation of the CP may not fully capture its complex morphology; nevertheless, given the large sample size, we relied on validated parcellation algorithms. Second, our findings require confirmation in large‐scale multicenter cohorts, and complementary animal experiments are needed to clarify the mechanistic basis of CP alterations in PD. Third, although we additionally adjusted all DTI‐ALPS‐related analyses for global white matter FA and MD to reduce the influence of generalized white matter microstructural degeneration, the DTI‐ALPS index remains a proxy marker and may still be affected by unmeasured factors. In particular, detailed vascular imaging or vascular risk data were not available in the present cohort, and the potential influence of vascular health on the ALPS index could therefore not be fully evaluated. Fourth, the cross‐sectional design precludes definitive conclusions about causality or the temporal sequence of the observed changes. Although our mediation analysis provides a statistical framework to explore potential pathways linking CP microstructure, ALPS index, and CC integrity, it cannot prove a causal relationship within a cross‐sectional cohort. Longitudinal studies will be essential to establish temporal relationships between CP alterations and disease progression. These limitations should be considered when interpreting our results, but they also highlight important avenues for future research.

In conclusion, this study suggests that CP microstructural alterations are associated with glymphatic dysfunction and CC abnormalities in PD. FW‐corrected diffusion imaging of the CP may provide useful information beyond volumetric measures alone and may serve as a complementary imaging marker for characterizing PD‐related pathological changes.

## Author Contributions

**Suyi Zhou** and **Zhiming Zhen:** writing – original draft, conceptualization, visualization, methodology. **Cheng Lai:** methodology, validation, data curation. **Peiyu Huang** and **Zhe Sun:** writing – review and editing, methodology, formal analysis. **Taotao Yang** and **Fengwei Yu:** contributed to data collection and statistical analysis. **Wei Chen (Eighth Author):** methodology; validation; writing – review and editing. **Zhentao Zuo:** writing – review and editing, supervision, resources. **Wei Chen (Tenth Author):** writing – review and editing, resources, supervision, funding acquisition.

## Funding

This work was supported by the Platform Construction Fund of the 7T Magnetic Resonance Imaging Translational Medicine Center of Southwest Hospital (No. 524Z2Q31); The Special Project for Enhancing Technological Innovation Capability of Army Medical University (No. 2023XQN31).

## Ethics Statement

The study was approved by the Ethics Committee of the First Affiliated Hospital of the Army Medical University (KY2023060). We confirm that the informed consent process was conducted in accordance with the ethical standards of the institutional review board and in compliance with the principles of the Declaration of Helsinki.

## Conflicts of Interest

The authors declare no conflicts of interest.

## Supporting information


**Table S1:** Demographic and clinical characteristics of patients with Parkinson's disease and healthy controls.
**Figure S1:** Flowchart of participants included in the study. HC, healthy controls; MRI, magnetic resonance imaging; PD, Parkinson's disease.
**Figure S2:** Representative placement of regions of interest for DTI‐ALPS index calculation. The axial image illustrates the locations of regions of interest in the projection fiber area (green) and association fiber area (yellow) used for DTI‐ALPS index calculation. DTI‐ALPS, diffusion tensor image analysis along the perivascular space.
**Figure S3:** Group comparisons of CP diffusion metrics between PD and HC participants. Violin‐box plots display group differences in CP diffusion parameters, including: (A) CP FW value; (B) CP FA_FWcorr_ and (C) CP_MDFWcorr_. *p < 0.05. PD, Parkinson's disease; HC, healthy controls; CP, choroid plexus; FW, free water; FA_FWcorr_, FW–corrected FA; MD_FWcorr_, FW–corrected MD.

## Data Availability

The data that support the findings of this study are available from the corresponding author upon reasonable request.
