# Supplementary material for: Choroid Plexus Microstructural Alterations Link Glymphatic Dysfunction and White Matter Degeneration in Parkinson's Disease
Source: CNS Neurosci Ther. 2026 Sep 6;32(9):e71039. doi: 10.1002/cns.71039 (PMC13547849; doi:10.1002/cns.71039)
Supplement: Supplementary file 1 [file CNS-32-e71039-s001.docx]

**Supplementary appendix**

**Supplementary Methods**

**Supplementary Method 1. DTI-ALPS analysis**

**Supplementary Method 1. DTI-ALPS analysis**

The DTI-ALPS index was calculated according to the method described by Taoka et al[1]. Anatomically, alongside the lateral ventricle bodies, the perivascular spaces are oriented along the right-left axis (x-axis), which runs orthogonal to the ventricular wall. Within this exact plane, the major projection fiber tracts are aligned along the head-foot axis (z-axis), and the major association fibers course along the anterior-posterior axis (y-axis). Because the perivascular spaces intersect these massive white matter tracts perpendicularly, quantifying water diffusivity strictly along the x-axis provides a targeted surrogate measure for perivascular diffusivity.

As depicted in Figure S2, 5-mm cross-shaped regions of interest (ROIs) were defined using an atlas-guided approach to localize the projection and association fiber regions adjacent to the lateral ventricles, consistent with previous implementations of the DTI-ALPS method[2]. The ROI positions were then manually checked for each individual on color-coded FA maps to ensure accurate placement and to avoid contamination from cerebrospinal fluid or gray matter. The formula is expressed as:

$$ALPS Index=\frac{mean(D_{xxproj},D_{xxassoc})}{mean(D_{yyproj},D_{zzassoc})}$$

The numerator represents the mean x-axis diffusivity in the projection and association fiber regions, and the denominator represents the mean diffusivities orthogonal to the dominant fiber directions in these respective regions. The final ALPS index for each participant was calculated as the average of the values obtained from both hemispheres.

**References：**

[1]	T. Taoka, Y. Masutani, H. Kawai, T. Nakane, K. Matsuoka, F. Yasuno, T. Kishimoto, S. Naganawa, Evaluation of glymphatic system activity with the diffusion MR technique: diffusion tensor image analysis along the perivascular space (DTI-ALPS) in alzheimer’s disease cases, Jpn. J. Radiol. 35 (2017) 172–178. https://doi.org/10.1007/s11604-017-0617-z.

[2]	X. Liu, G. Barisano, X. Shao, K. Jann, J.M. Ringman, H. Lu, K. Arfanakis, A. Caprihan, C. DeCarli, B.T. Gold, P. Maillard, C.L. Satizabal, E. Fadaee, M. Habes, L. Stables, H. Singh, B. Fischl, A. van der Kouwe, K. Schwab, K.G. Helmer, S.M. Greenberg, D.J.J. Wang, MarkVCID Consortium, Cross-vendor test-retest validation of diffusion tensor image analysis along the perivascular space (DTI-ALPS) for evaluating glymphatic system function, Aging Dis. 15 (2024) 1885–1898. https://doi.org/10.14336/AD.2023.0321-2.

**Part Ⅱ. Supplementary Table and Figures**

**Supplementary Table 1. Demographic and Clinical Characteristics of Patients with Parkinson’s Disease and Healthy Controls**

**Supplementary Figure 1. Flowchart of participants included in the study**

**Supplementary Figure 2. Representative placement of regions of interest for DTI-ALPS index calculation**

**Supplementary Figure 3. Group comparisons of CP diffusion metrics between PD and HC participants**

**Supplementary Table 1** Demographic and Clinical Characteristics of Patients with Parkinson’s Disease and Healthy Controls

|  | PD (*n* = 81) | HC (*n* = 80) | *t*/*χ^2^* | *p* value |
| --- | --- | --- | --- | --- |
| Age (years) | 62.27 ± 7.81 | 61.99 ± 6.21 | 0.25 | 0.80 |
| Sex (male, %) | 38 (46.91%) | 31(38.75%) | 1.10 | 0.30 |
| Disease duration (years) | 6.10 ± 4.17 | - | - | - |
| Education (years) | 9.82 ± 3.46 | 12.79 ± 2.80 | −5.93 | < 0.001*** |
| LEDD (mg/day) | 543.75 (375.00,675.00) | - | - | - |
| UPDRS Ⅲ | 33.45 ± 16.91 | - | - | - |
| H-Y stage | 2.19 ± 0.76 | - | - | - |
| MMSE | 26.58 ± 2.72 | 28.32 ± 1.91 | −4.46 | < 0.001*** |
| HAMD | 13.69 ± 9.04 | 3.99±3.71 | 8.44 | < 0.001*** |

MMSE, Mini-Mental State Examination; PD, Parkinson’s disease; HC, healthy controls; LEDD, levodopa equivalent daily dose; UPDRS III, Unified Parkinson’s Disease Rating Scale Part III; H-Y stage, Hoehn and Yahr stage; HAMD, Hamilton Depression Rating Scale. Continuous variables are presented as mean ± standard deviation or median (interquartile range), as appropriate. Group comparisons were performed using independent-samples t tests or chi-square tests, as appropriate. ****p* < 0.001.

**Supplementary Figure 1. Flowchart of participants included in the study.** MRI, magnetic resonance imaging; PD, Parkinson’s disease; HC, healthy controls.

**Supplementary Figure 2. Representative placement of regions of interest for DTI-ALPS index calculation.** The axial image illustrates the locations of regions of interest in the projection fiber area (green) and association fiber area (yellow) used for DTI-ALPS index calculation. DTI-ALPS, diffusion tensor image analysis along the perivascular space.

**Supplementary Figure 3. Group comparisons of CP diffusion metrics between PD and HC participants.** Violin-box plots display group differences in CP diffusion parameters, including:(A) CP FW value; (B) CP FA_FWcorr_ and (C) CP _MDFWcorr_. * *p* < 0.05. PD, Parkinson’s disease; HC, healthy controls; CP, choroid plexus; FW, free water; FA_FWcorr_, FW–corrected FA; MD_FWcorr_, FW–corrected MD.
